# Clinical cognition in the age of cardiovascular AI

**DOI:** 10.3389/fcvm.2026.1872551

**Published:** 2026-07-16

**Authors:** Jose E. Krieger

**Affiliations:** Instituto do Coração (InCor), Hospital das Clinicas HCFMUSP, Faculdade de Medicina, Universidade de Sao Paulo, Sao Paulo, Brazil

**Keywords:** artificial intelligence, automation bias, cardiovascular medicine, clinical cognition, decision support, explainable AI, health equity, implementation science

## Abstract

Artificial intelligence (AI) is rapidly entering cardiovascular medicine through electrocardiography, imaging, wearable monitoring, risk prediction, heart failure management, and clinical decision support. Its value, however, should not be judged only by technical accuracy, speed, or computational sophistication. Cardiovascular care requires clinicians and teams to convert multimodal, longitudinal, incomplete, and context-dependent information into action under uncertainty. This Perspective argues that AI should be understood not as a replacement for clinical intuition, but as a cognitive instrument that reshapes how cardiovascular teams perceive, prioritize, reason, decide, communicate, and learn. Building on dual-process theories of clinical reasoning, the manuscript proposes that AI can support both rapid pattern recognition (System 1) and slower analytic reasoning (System 2), while also creating new vulnerabilities when automation bias, alert fatigue, poor explainability, dataset shift, hidden inequity, or responsibility drift distort judgment. The central standard should therefore move from algorithm-centered performance to accountable intelligence: AI that is accurate, explainable, locally validated, equitable, auditable, monitored across its lifecycle, and embedded within explicit clinical governance.

## Introduction

### The missing question in cardiovascular AI

Artificial intelligence is now being applied across cardiovascular medicine, including electrocardiographic interpretation, echocardiography, cardiac computed tomography, cardiac magnetic resonance imaging, wearable monitoring, arrhythmia detection, heart failure management, coronary disease assessment, risk prediction, and clinical decision support. Much of the debate has appropriately focused on algorithmic performance: discrimination, calibration, diagnostic accuracy, segmentation quality, image interpretation, and workflow efficiency. These metrics are necessary, but incomplete, since cardiovascular care is not simply a prediction task. It is a cognitive, team-based, longitudinal, and ethically accountable process through which incomplete and heterogeneous information is transformed into clinical action.

The central question is therefore not only whether AI can predict cardiovascular outcomes, but whether AI improves cardiovascular thinking. A technically strong model may still be clinically weak if it distracts attention, hides uncertainty, fragments responsibility, performs poorly in local populations, or generates recommendations that cannot be acted upon. Conversely, a modest model may be valuable if it helps a team identify a patient who would otherwise be missed, retrieve the relevant longitudinal pattern, or make uncertainty explicit at the moment of decision.

Clinical intuition is rapid, experience-based pattern recognition. It allows experienced clinicians, nurses, sonographers, monitoring technicians, and other professionals to recognize instability, deterioration, or a familiar phenotype before all analytic steps are consciously articulated. Dual-process theories distinguish this fast intuitive mode, often described as System 1, from slower analytic reasoning, often described as System 2, while emphasizing that expert judgment depends on their interaction rather than on either mode alone (1). Cognition is broader than intuition. It includes perception, attention, memory, representation, reasoning, executive control, learning, decision-making, metacognition, and communication. These functions are not located only inside an individual physician. In contemporary cardiovascular care, they are distributed across clinicians, patients, families, electronic health records, laboratories, imaging systems, monitors, protocols, guidelines, institutions, and now AI systems.

Throughout this Perspective, accountable intelligence denotes AI that is not only statistically accurate, but clinically answerable. An accountable AI system has a defined intended use, produces outputs that can be interpreted in context, displays relevant uncertainty and limitations, supports a plausible action pathway, allows human override, is locally validated and calibrated, is assessed for inequitable performance, is monitored after deployment, and is governed by explicit institutional responsibility. In this sense, accountability is not a property of the algorithm alone. It is a property of the full sociotechnical system in which an algorithm, interface, clinical team, patient, institution, and regulatory environment interact.

**Figure 1:**
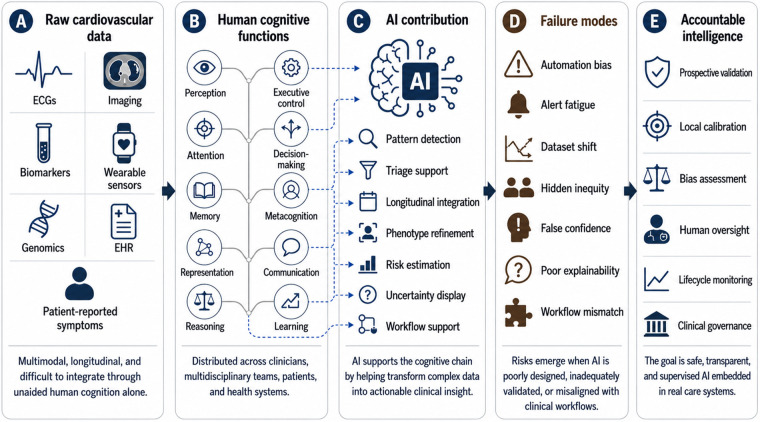
The cognitive chain of AI-enabled cardiovascular care. Artificial intelligence should be understood not as an autonomous replacement for clinical judgment, but as a cognitive instrument embedded within cardiovascular care. Heterogeneous raw data streams, including ECGs, imaging, biomarkers, wearable sensors, genomics, electronic health records, and patient-reported symptoms, enter a distributed cognitive system involving clinicians, multidisciplinary teams, patients, institutions, and health systems. AI may contribute to System 1 cognition by enhancing pattern detection, salience, triage, and early recognition; it may contribute to System 2 cognition by supporting longitudinal integration, phenotype refinement, analytic reasoning, uncertainty display, workflow support, and learning. Failure modes include automation bias, alert fatigue, dataset shift, hidden inequity, false confidence, poor explainability, workflow mismatch, and responsibility drift. Accountable intelligence requires prospective validation, local calibration, bias assessment, human oversight, lifecycle monitoring, role-specific training, and clinical governance. The central measure of success is whether AI improves how cardiovascular teams perceive, prioritize, reason, decide, communicate, and learn.

For this reason, cardiovascular AI should be framed less as a rival to clinical judgment and more as a cognitive technology. Like echocardiography, biomarkers, catheterization, and genomic testing, AI changes what clinicians can perceive and how they reason. Unlike many traditional tools, however, AI may also shape attention, confidence, triage, communication, responsibility, and institutional behavior. This is why cardiovascular AI must be designed and evaluated not only for prediction, but for judgment (Figure 1).

### Why cardiovascular medicine is a cognitive stress test

Cardiovascular medicine is an ideal field in which to examine AI as a cognitive instrument because it combines acute time pressure, chronic disease, quantitative imaging, physiological reasoning, procedural decisions, pharmacological complexity, device therapy, genetics, prevention, and population health. A patient with dyspnea may require simultaneous consideration of heart failure, ischemia, arrhythmia, pulmonary embolism, renal dysfunction, anemia, infection, valvular disease, medication effects, frailty, and social vulnerability. A patient with cardiomyopathy may require integration of electrocardiography, echocardiography, cardiac magnetic resonance, family history, genotype, biomarkers, arrhythmia burden, exercise capacity, and treatment response.

This complexity explains why AI should not be evaluated only as a diagnostic classifier. It should be evaluated as an intervention in the reasoning process. Does it help the team see earlier, prioritize better, remember more, represent disease more accurately, reason more transparently, act more safely, and learn from outcomes? Recent statements and reviews have described the expanding role of AI in cardiovascular diagnosis, imaging, monitoring, risk assessment, and workflows (2–4). The next step is to ask whether these tools improve the cognitive architecture of care.

Consider heart failure. A model that predicts hospitalization risk is useful only if it helps distinguish among congestion, arrhythmia, renal dysfunction, infection, medication gaps, nonadherence, progressive myocardial disease, frailty, and socioeconomic barriers. The same predicted risk may require very different actions depending on the mechanism. Similarly, an ECG-based AI tool that detects occult left ventricular dysfunction improves care only if it identifies patients who would otherwise be missed, triggers appropriate echocardiography, avoids excessive false positives, performs well across sex, age, ancestry, and comorbidity groups, and remains accurate after changes in devices, data flow, and local patient populations.

### The dual-process cognitive chain of AI-enabled cardiovascular care

A cognition-centered view asks where in the clinical reasoning process AI intervenes. In dual-process terms, AI can modify the cues that feed rapid System 1 recognition and the evidence base used for slower System 2 analysis. This distinction is clinically important. If an ECG algorithm highlights a subtle occlusion-myocardial-infarction pattern, it may accelerate System 1 recognition by making a previously hidden perceptual cue salient. If a heart-failure dashboard reconstructs a six-month trajectory of weight, natriuretic peptides, renal function, medication titration, device alerts, and admissions, it supports System 2 reasoning by organizing evidence that would otherwise exceed working memory. A safe system should strengthen the dialogue between these modes rather than allow either to dominate uncritically.

Perception is among the most mature domains. AI can extract high-dimensional signals from ECGs, imaging, photoplethysmography, radiographs, and magnetic resonance images, including patterns that may be subtle or inconsistently recognized by humans (5–11). Yet perception is not diagnosis. The AI-enhanced ECG or image should be a richer perceptual gateway to targeted evaluation, not an autonomous conclusion. In echocardiography or cardiac magnetic resonance, automated quantification may improve consistency and speed, but clinical value depends on performance in real patients, including those with poor acoustic windows, arrhythmias, prosthetic valves, congenital abnormalities, or complex loading conditions.

Attention is equally important. Cardiovascular teams face continuous information overload from telemetry, laboratories, imaging reports, device alerts, remote monitoring streams, electronic messages, and symptoms. AI can help by identifying patients at risk and directing scarce clinical attention to the right problem at the right time. However, attention tools act directly on System 1 salience: they can make a patient look more urgent, less urgent, or more certain than the underlying evidence warrants. Poorly calibrated attention tools can therefore create alert fatigue or automation bias, in which users over-rely on automated recommendations even when they are incomplete, uncertain, or wrong.

Memory and representation may be among the most underappreciated contributions of AI. Human memory is not designed to retain every prior ECG, echocardiogram, medication change, hospitalization, biomarker trajectory, genetic variant, and family history. Cardiovascular disease is often longitudinal, and clinical meaning frequently depends on change over time. AI-supported records could summarize serial ejection fraction, ventricular volumes, arrhythmia burden, renal function, natriuretic peptides, medication titration, family screening, cascade testing, and variant reinterpretation. The goal is not more data; it is the right prior context at the point when it changes the next decision.

Representation determines what problem is being solved. The same patient may be represented as having dyspnea, heart failure with preserved ejection fraction, atrial fibrillation with congestion, ischemic heart disease, obesity-related cardiometabolic disease, amyloidosis, valvular disease, or frailty with cardiovascular vulnerability. A useful AI system should help refine these representations rather than compress complexity into an unexplained risk score. In coronary disease, for example, AI-derived plaque quantification may add value beyond stenosis severity, but it must be interpreted together with symptoms, ischemia, diabetes, LDL exposure, inflammation, treatment adherence, and patient preference.

Reasoning connects evidence to inference, and prediction is not explanation. Knowing that a patient is at high risk is less useful than understanding whether that risk is driven by congestion, ischemia, arrhythmia, fibrosis, inflammation, renal dysfunction, frailty, treatment gaps, or social determinants. Explainability is therefore not a decorative feature; it is part of confidence calibration, clinical communication, accountability, and learning. The right explanation depends on the user and the moment. A cardiologist may need variables, uncertainty, model limits, and guideline context. A nurse may need an actionable threshold and escalation pathway. A monitoring technician may need to know whether an alert reflects signal artifact or physiological deterioration. A patient may need a clear reason for the next step.

This argument should not be treated as a purely conceptual postulate. Recent cardiovascular adoption studies suggest that explanation, training, and workflow integration materially influence trust and use. A large French multiprofessional cardiovascular survey reported that only 7.8% of respondents had formal AI training and that allied-health professionals were more often non-users than cardiologists; confidence in AI outputs increased substantially when an explanatory rationale was provided, reaching 84% among cardiologists and 77% among allied-health and other professionals (12). Earlier mixed-methods work among cardiologists and health-information-technology administrators identified limited knowledge, insufficient usability, cost constraints, poor electronic-health-record interoperability, and lack of trust as major adoption barriers (13). A broader cardiovascular mixed-methods study similarly identified transparency, accountability, infrastructure, change management, and acceptance as implementation challenges (14). Together, these data support a practical interpretation of explainability: it should help users calibrate trust, detect when the model is outside its intended use, and translate the output into safe action.

Executive control and metacognition are the safeguards that prevent AI from becoming an oracle. Executive functions include inhibitory control, working memory, and cognitive flexibility (15, 16). In AI-enabled care, they require clinicians and teams to ask whether the data are reliable, whether the patient is within the model's intended use, whether important variables are missing, whether the output should change management, and who is responsible for acting. If a model assigns low risk to a patient with ongoing chest pain, dynamic ECG changes, hypotension, or high clinical suspicion, executive control requires resisting the model and escalating care. Oversight is therefore active cognitive control, not passive approval.

### How AI can make cognition worse

A cognition-centered framework also clarifies why AI can fail even when the model appears technically strong. The danger is not only that AI may be wrong; the deeper danger is that it may change how humans think when it is wrong, uncertain, or misapplied. It can make the irrelevant look urgent, the uncertain look definitive, and the local context look unnecessary.

Several failure modes are especially relevant in cardiovascular medicine. Automation bias occurs when users over-trust the model (17). Alert fatigue occurs when excessive warnings dilute attention. False confidence occurs when a polished interface hides uncertainty. Dataset shift occurs when a model is moved into a different population, device, assay, imaging protocol, coding practice, or workflow. Hidden inequity occurs when performance differs across sex, age, ancestry, socioeconomic context, device quality, or comorbidity. Responsibility drift occurs when everyone assumes that someone else checked the output.

These risks are not hypothetical, algorithmic bias has been shown to arise when apparently neutral proxies encode unequal access to care (18). In cardiovascular medicine, similar risks may emerge if models are trained on data that reflect unequal referral patterns, imaging quality, follow-up access, medication availability, coding practices, or ancestry representation. A coronary risk model developed in predominantly European-ancestry populations may miscalibrate in admixed populations. A model trained in a tertiary hospital may fail in primary care. A tool developed with high-quality imaging may perform poorly with lower-quality acquisitions.

Dual-process theory helps explain why these failures can be powerful. A high-risk flag may capture attention before analytic reasoning begins. A low-risk label may prematurely close diagnostic search. A confident-looking dashboard may suppress doubt. A model explanation may appear mechanistic even when it is only associational. The goal is not to force clinicians to ignore AI; it is to design systems that create appropriate friction at moments of uncertainty, mismatch, or high consequence.

### From algorithm-centered evaluation to accountable intelligence

Most AI evaluation begins with technical validity, that remains essential, but it is not enough. Cardiovascular AI should be evaluated as a clinical-cognitive intervention. A model that improves the area under the curve but worsens workflow, equity, communication, or actionability has not improved care. Conversely, a model with moderate discrimination may be valuable if it identifies a treatable failure mode, reduces delays, or improves team coordination.

Accountable intelligence requires several linked forms of evidence. Technical validity asks whether the model performs statistically, including discrimination, calibration, sensitivity, specificity, uncertainty, and subgroup performance. Clinical validity asks whether the output detects or predicts something meaningful and actionable. Cognitive validity asks whether the model improves interpretation, attention, reasoning, confidence calibration, and error detection. Workflow validity asks whether the team can act safely on the output, with acceptable alert burden, time-to-action, documentation, and escalation pathways. Equity validity asks whether performance is reliable across relevant populations and settings. Lifecycle validity asks whether the model remains safe after deployment.

Regulators and health systems increasingly emphasize transparency, validation, governance, monitoring, and change-control planning for AI-enabled medical software (19, 20). For cardiovascular teams, the practical implication is straightforward: model approval is not the end of evaluation; it is the beginning of local responsibility. Silent trials, prospective validation, local calibration, bias assessment, drift monitoring, documentation of decisions, retraining rules, and retirement criteria should be considered part of the technology. Vendor validation is not the same as local safety.

Accountability also requires traceable responsibility. Before deployment, institutions should specify who receives the output, who must acknowledge it, what action is expected, when escalation is required, how disagreement is documented, how overrides are reviewed, and when the model should be paused or retired. Without this operational definition, accountability becomes a moral aspiration rather than a property of clinical care.

### Multiprofessional cognition, training, and social validation

A cognition-centered framework cannot be physician-only. Cardiovascular care is multiprofessional: nurses detect deterioration, titrate therapies, and coordinate follow-up; sonographers and imaging technicians acquire the data on which AI depends; monitoring teams triage device and wearable alerts; pharmacists identify interactions and adherence barriers; genetic counselors communicate inherited risk; administrators and information-technology teams determine whether the tool is usable, interoperable, and auditable. Each group interacts with AI at a different point in the cognitive chain and therefore needs different explanations, thresholds, training, and governance.

This professional heterogeneity is empirically important. In the French multiprofessional cardiovascular survey, cardiologists, allied-health professionals, and other professionals differed in AI use, exposure, trust, performance expectations, and declared training needs (12). Allied-health professionals were more often non-users of AI tools and chatbots, and they reported different performance thresholds and training preferences. These findings imply that a single interface, a single educational module, or a single trust message is unlikely to be adequate. For a cardiologist, the central issue may be whether a model is calibrated and clinically actionable. For a sonographer, it may be whether AI improves acquisition quality without obscuring image limitations. For a nurse or monitoring technician, it may be whether an alert is actionable, urgent, or likely to be artifact.

Social influence should also be interpreted cognitively rather than superficially. AI adoption is not driven only by individual enthusiasm or peer imitation. Cardiovascular professionals appear to place particular weight on institutional, scientific, and guideline-based validation. In the French survey, adoption conditional on guideline endorsement was reported more frequently than adoption conditional on successful peer use (12). This matters because guidelines, institutional procurement, and local governance are forms of distributed cognition: they determine which tools are perceived as legitimate, when clinicians feel permitted to rely on them, and how responsibility is shared.

Therefore, accountable AI should include role-specific training and socially explicit validation. Training should teach users not only what the model predicts, but what data it uses, what it omits, how it fails, how uncertainty is displayed, when to override it, and how to document disagreement. Institutional endorsement should be accompanied by local performance monitoring and clear escalation rules, so that social trust does not become uncritical trust.

### Practical implications for clinicians, developers, and institutions

A cognition-centered framework translates into practical responsibilities. Clinicians should cultivate calibrated trust: neither reflexively accepting AI as a superior oracle nor dismissing it because it is not human. The appropriate question at the bedside is whether the output fits the patient, the physiology, the available evidence, and the intended use. When it does, AI can support earlier detection, prioritization, and communication. When it does not, clinicians must be able to override it, document the reasoning, and learn from the discrepancy.

Developers should design for the moment of use. A risk score without a plausible action pathway is often an information burden rather than a cognitive aid. The output should specify what was detected or predicted, which data drove the result, what important data were missing, how uncertain the estimate is, whether the patient resembles the validation population, what actions may reasonably follow, and what actions should not follow. Interfaces should make uncertainty visible rather than hiding it behind polished dashboards.

Institutions should treat AI deployment as a clinical governance responsibility. Local validation, escalation pathways, documentation standards, role-specific user training, equity audits, monitoring dashboards, and model retirement criteria should be specified before routine use (21–23). Patients and families also need understandable communication: what the AI result means, what it does not mean, and how it will influence the next decision.

### Equity, local calibration, and the cardiovascular AI divide

Cardiovascular AI will not be equitable by default. Models reflect the data, devices, clinical labels, access patterns, and health systems from which they are built. This is particularly important for countries with admixed populations and heterogeneous access to care, such as Brazil. A model used in the public health system, private hospitals, remote monitoring programs, or genetic cardiology clinics should be tested in the populations and workflows where it will influence decisions. Otherwise, AI may widen the very gaps it promises to close.

Equity requires more than fairness metrics after deployment. It requires representative data strategies, transparent reporting, subgroup calibration, prospective evaluation, and governance mechanisms that can detect when performance differs across populations or care settings. For cardiovascular AI, local calibration is not a secondary implementation detail; it is part of scientific validity.

### Discussion: from artificial intelligence to accountable intelligence

The future of cardiovascular AI should not be framed as a competition between clinicians and machines. The better question is whether AI can improve the cognitive architecture of cardiovascular care. The best systems will not simply classify images, predict events, or generate recommendations. They will help teams perceive earlier, prioritize better, remember longitudinally, represent disease more accurately, reason more transparently, decide more safely, communicate more clearly, and learn continuously.

This perspective avoids two common mistakes. The first is technological overconfidence: assuming that better prediction automatically means better care. The second is defensive skepticism: assuming that because AI is not human, it cannot meaningfully support human judgment. Both positions misjudge the technology. AI is not intuition, wisdom, empathy, responsibility, or clinical experience, but it can change what humans see, remember, compare, question, and decide. That is why the appropriate standard is not replacement, but accountable augmentation.

The revision proposed here also makes a stronger theoretical claim: cardiovascular AI should be designed to improve the interaction between System 1 and System 2 reasoning. It should make hidden patterns perceptible without making them falsely definitive; it should support analytic reasoning without overwhelming clinicians with uninterpretable data; it should increase confidence when evidence is strong and preserve doubt when evidence is weak or context is mismatched. In this sense, accountable intelligence is not only safer AI. It is better clinical cognition.

Cardiovascular medicine should move from algorithm-centered AI to cognition-centered AI: systems designed not only to be accurate, but also to make care more thoughtful, equitable, explainable, and safe. The relevant comparison is not AI vs. physician. It is usual care vs. a redesigned care process in which clinicians, patients, teams, institutions, and AI systems together make better decisions than any of them could make alone (Table 1).

**Table 1:** A cognition-centered evaluation framework for cardiovascular AI.

Evaluation level	Core question	Suggested evidence
Technical validity	Does the model perform statistically?	Discrimination, calibration, sensitivity, specificity, uncertainty, and subgroup performance
Clinical validity	Does it detect or predict something meaningful and actionable?	Association with outcomes, comparison with standard care, clinical utility, decision curves, and prospective validation
Dual-process cognitive validity	Does it improve the interaction between rapid recognition and analytic reasoning?	User testing, interpretation studies, impact on attention, reasoning, confidence calibration, diagnostic closure, and error detection
Multiprofessional validity	Does it support the roles that actually use or act on the output?	Role-specific testing among cardiologists, nurses, sonographers, technicians, pharmacists, genetic counselors, IT teams, and administrators
Workflow and social validity	Can the team act on it safely within local routines and institutional expectations?	Silent trials, implementation pilots, alert burden, time-to-action, documentation quality, escalation pathways, and guideline/institutional endorsement
Equity validity	Does it work across populations and care settings?	Sex, age, ancestry, socioeconomic, device, comorbidity, acquisition-quality, and local calibration analyses
Lifecycle and governance validity	Does it remain reliable and accountable after deployment?	Monitoring dashboards, drift detection, audit trails, retraining rules, change-control plans, override review, and retirement criteria

## Data Availability

The original contributions presented in the study are included in the article/Supplementary Material, further inquiries can be directed to the corresponding author.
